# *Polygonum hydropiper* Compound Extract Inhibits *Clostridium perfringens*-Induced Intestinal Inflammatory Response and Injury in Broiler Chickens by Modulating NLRP3 Inflammasome Signaling

**DOI:** 10.3390/antibiotics13090793

**Published:** 2024-08-23

**Authors:** Jinwu Zhang, Chunzi Peng, Maojie Lv, Shisen Yang, Liji Xie, Jiaxun Feng, Yingyi Wei, Tingjun Hu, Jiakang He, Zhixun Xie, Meiling Yu

**Affiliations:** 1 Guangxi Key Laboratory of Animal Breeding, Disease Control and Prevention, College of Animal Science and Technology, Guangxi University, Nanning 530004, China; 2018302041@st.gxu.edu.cn (J.Z.); 2218393073@st.gxu.edu.cn (S.Y.);; 2 Guangxi Key Laboratory of Veterinary Biotechnology, Guangxi Veterinary Research Institute, Nanning 530001, China; 3 College of Life Science and Technology, Guangxi University, Nanning 530004, China

**Keywords:** *Polygonum hydropiper* compound extract, *Necrotic enteritis*, network pharmacology, oxidative stress, NLRP3 inflammasome

## Abstract

*Necrotic enteritis* (*NE*) is a critical disease affecting broiler health, with *Clostridium perfringens* as its primary pathogen. *Polygonum hydropiper* compound extract (PHCE), formulated based on traditional Chinese veterinary principles, contains primarily flavonoids with antibacterial, anti-inflammatory, and antioxidant properties. However, PHCE’s efficacy against *Clostridium perfringens*-induced *NE* and its underlying mechanism remain unclear. This study employed network pharmacology and molecular docking to predict PHCE’s potential mechanisms in treating NE, followed by determining its minimum inhibitory concentration (MIC) and minimum bactericidal concentration (MBC) against *Clostridium perfringens* (*C. perf*). Subsequently, the effects of various PHCE doses on intestinal damage, antioxidant capacity, and inflammatory factors in *C. perf*-infected broilers were assessed. Network pharmacology and molecular docking suggested that PHCE’s therapeutic mechanism for *NE* involves the NOD-like receptor thermal protein domain associated protein 3 (NLRP3) inflammasome signaling pathway, with flavonoids such as quercetin, kaempferol, and isorhamnetin as key active components. PHCE exhibited an MIC of 3.13 mg/mL and an MBC of 12.5 mg/mL against *C. perf*. High PHCE doses effectively reduced intestinal damage scores in both the jejunum and ileum, accompanied by attenuated intestinal pathological changes. Additionally, the high dose significantly increased superoxide dismutase (SOD) levels while decreasing malondialdehyde (MDA), hydrogen peroxide (H_2_O_2_), tumor necrosis factor-alpha (TNF-α), interleukin-1 beta (IL-1β), and interleukin-6 (IL-6) in the jejunum and ileum (*p* < 0.01 or *p* < 0.05). PHCE also modulated the expression of caspase-1, IL-1β, gasdermin D (GSDMD), and NLRP3 mRNA, key components of the NLRP3 inflammasome signaling pathway, in both intestinal segments. These findings collectively indicate that PHCE protects against *C. perf*-induced oxidative stress and inflammatory damage in *NE*. By enhancing antioxidant capacity, PHCE likely reduces oxidative stress and inflammatory responses, subsequently modulating NLRP3 inflammasome signaling pathway key factor expression. Overall, this research provides valuable insights into the protective mechanism of the herbal compound PHCE and its potential benefits for avian health.

## 1. Introduction

*Necrotic enteritis* (*NE*) is a significant intestinal disease in poultry, manifesting in either acute clinical or chronic subclinical forms. The latter is prevalent in the global poultry industry, representing a substantial threat to broiler production and resulting in approximately $6 billion in economic losses annually [1]. *NE* is caused by pathogenic *Clostridium perfringens* (*C. perf*), a spore-forming, anaerobic, Gram-positive bacterium, which can also be a component of the normal microbiota in humans and animals [2,3]. The pathogenesis of *NE* is primarily attributed to toxins produced by *C. perf*, including α-toxin (CPA), β-toxin (CPB), ε-toxin (ETX), ι-toxin (ITX), enterotoxin (CPE), and NetB toxin (NetB) [4]. The conserved *CPA* gene encodes phospholipase C, sphingomyelinase, and a zinc-dependent metalloenzyme with hemolytic activity. This metalloenzyme is capable of hydrolyzing phospholipids within cell membranes, thereby inducing membrane dysfunction and subsequent cell death [2]. Known for its hemolytic, skin necrotic, and lethal properties, CPA plays a pivotal role in gangrene [5]. While PFO is leukotoxic at high doses, it stimulates the production of intracellular adhesion molecule 1 (ICAM-1) and adhesion glycoprotein CD11b/CD18 in endothelial cells at sublethal concentrations, resulting in leukocyte stasis in vessels adjacent to gangrene [6,7]. *C. perf* types A and C are the primary causative agents of *NE* in poultry, with type A strains producing toxins such as CPA and PFO [8]. Although *C. perf* exhibits low pathogenicity in the intestinal tract of healthy poultry, environmental changes or feed-induced stress can trigger its overgrowth, leading to food poisoning, *NE*, and other diseases [9]. Antibiotics were once the mainstay of *NE* treatment, but concerns over residues, contamination, and resistance have prompted their ban in many countries. Consequently, *NE* cases surged, necessitating urgent development of antibiotic alternatives [10]. Traditional Chinese medicine has emerged as a promising replacement due to its low toxicity, minimal residue, and reduced likelihood of inducing drug resistance [11].

In recent years, clinical applications of *Polygonum hydropiper* L. and its compounds have steadily increased, with studies demonstrating their efficacy against various bacterial and fungal diseases. Extracts of *Polygonum hydropiper* L. exhibit protective effects against *Vibrio parahaemolyticus*-infected *Litopenaeus vannamei*, and the minimum inhibitory concentration (MIC) against *Aeromonas hydrophila*, a pathogen affecting weather fish, is less than 10 μL/mL, indicating potent bacteriostatic activity [12,13]. *Polygonum hydropiper* compound extract (PHCE) is formulated based on traditional Chinese veterinary medicine principles, incorporating six herbs, including *Polygonum hydropiper* L. and patchouli. As the core component, *Polygonum hydropiper* L., with its heat-clearing and detoxifying properties, constitutes the largest proportion of PHCE and plays a primary role. The remaining herbs—*Patchouli*, *Pulsatilla*, *Astragalus*, *Ligustrum lucidum*, and *licorice*—enhance the therapeutic efficacy of *Polygonum hydropiper* L. while mitigating potential adverse effects [14]. PHCE, a commonly used herb in Guangxi, contains abundant flavonoids and is employed in treating dysentery, gastroenteritis, diarrhea, and other gastrointestinal disorders. Plant-derived flavonoids possess diverse biological activities, including anti-inflammatory, antiviral, antioxidant, and antimicrobial properties [15,16,17]. Liu et al. demonstrated that licorice chalcone A, extracted from licorice, reduced pro-inflammatory TNF-α levels in broiler chicken small intestinal tissues and effectively treated *C. perf*-induced *NE* with associated intestinal pathology [18]. In addition, the traditional Chinese medicine compound composed of *Polygonum hydropiper* L. also has great efficacy in treating animal diseases. Shen reported the efficacy of a compound *Polygonum hydropiper* powder containing *Polygonum hydropiper* L., cinnabar, and tea in preventing and treating contagious porcine gastroenteritis [19].

Network pharmacology, a novel discipline rooted in systems biology, analyzes biological system networks to identify specific signaling nodes for targeted drug molecule design [20]. Its aim is to systematically elucidate complex scientific challenges at multiple levels [21]. A key characteristic of network pharmacology is its predictive nature, emphasizing multi-channel signaling pathway regulation to enhance therapeutic efficacy, reduce adverse effects, and improve clinical trial success rates, thereby lowering drug research and development costs [22]. Molecular docking, a drug design method based on receptor characteristics and drug–receptor interactions, is a theoretical simulation technique for predicting binding patterns and affinities. It has become a crucial technology in computer-aided drug research [23]. To optimize the development and utilization of PHCE, this study integrated network pharmacology and molecular docking to explore potential network targets and mechanisms of action in treating chicken *NE*. PHCE and its constituents were extracted using decoction and alcohol extraction methods and subsequently applied to *C. perf* type A. The in vitro bacteriostatic effects of PHCE on *C. perf* type A were evaluated by determining the MIC, minimum bactericidal concentration (MBC), and bacteriostatic curves. To further elucidate PHCE’s protective effects and mechanisms against *NE* in vivo, different PHCE doses were incorporated into broiler chicken diets. This experimental approach investigated PHCE’s protective effects and mechanisms in the jejunum and ileum, providing a theoretical foundation for PHCE application in preventing and controlling *C. perf*-induced *NE* in broilers.

## 2. Results

### 2.1. Compound-Target Network

**Figure 1:**
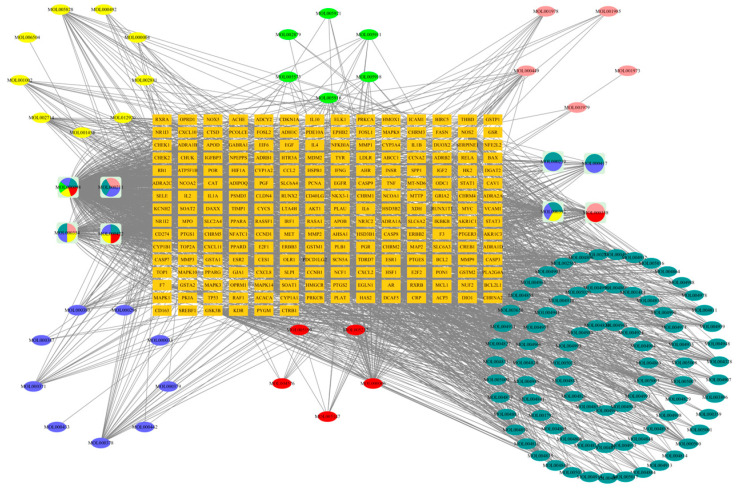
Herb–compound–target network of PHCE. Nine yellow ellipses and four yellow sectors represent *Polygonum hydropiper* L. components, six green ellipses and one green sector represent patchouli components, five pink ellipses and three pink sectors represent pulsatilla components, nine blue ellipses and seven blue sectors represent astragalus components, five red ellipses and thee red sectors represent *Ligustrum lucidum* components, and eighty-one cyan ellipses and seven cyan sectors represent licorice components. Orange rectangles represent the 216 potential targets of PHCE. Circles represent compounds shared among different herbs.

As illustrated in Figure 1, a Traditional Chinese Medicine Systems Pharmacology (TCMSP) search identified 140 active ingredients within PHCE: 13 from *Polygonum hydropiper* L., 7 from patchouli, 8 from pulsatilla, 16 from astragalus, 8 from *Ligustrum lucidum*, and 88 from licorice, along with 216 potential targets. The PHCE compound–target network, visualized in Cytoscape, comprised 339 nodes and 2291 edges. The top 10 drug components were selected based on degree, betweenness centrality, and closeness centrality, with higher degree values indicating greater network significance (Table 1). Within this network, active ingredients such as quercetin, kaempferol, isorhamnetin, and lignocerol exhibited high degree values, suggesting their potential as key components of PHCE for *NE* treatment.

### 2.2. Protein–Protein Interaction (PPI) Network Construction

**Figure 2:**
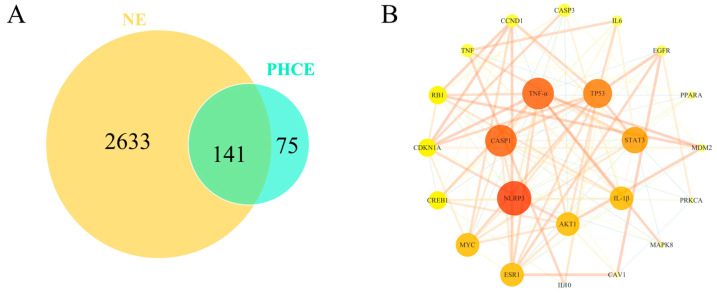
Network pharmacological analysis of PHCE and NE. (**A**) Venn diagram illustrating the overlap between PHCE targets and NE targets. (**B**) Protein–protein interaction (PPI) network of shared PHCE and NE targets. Nodes represent proteins, with a color gradient from yellow to red, indicating increasing protein interaction strength. Edges represent protein–protein association.

As shown in Figure 2A, PHCE exhibited 216 targets, while NE displayed 2774 targets, with 141 shared targets between the two. Figure 2B illustrates the PHCE and NE common target PPI network. In this network, node size and darkness correlated with degree value, indicating the likelihood of a target being a core target, such as NLRP3 and CASP1.

### 2.3. Results of Functional Enrichment Analysis

**Figure 3:**
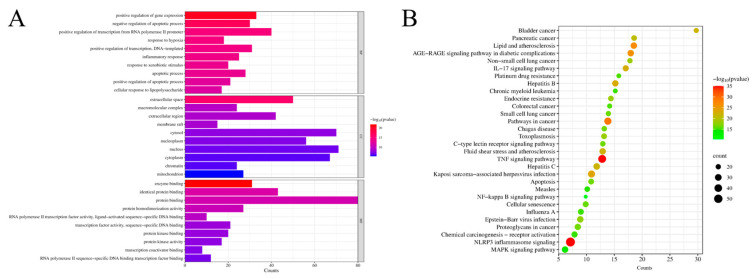
Network pharmacology prediction for PHCE treatment of *NE*. Top 10 GO terms of hub genes (**A**) and top 30 KEGG pathway of hub genes (**B**). The cut-off values of count and −log10(pvalue) for (**A**) are 5.3 and 8, respectively, while the cut-off values of count and −log10(pvalue) for (**B**) are 10.3 and 16, respectively.

As illustrated in Figure 3A, GO enrichment analysis identified 414 GO entries (*p* < 0.01), from which the top ten terms were selected for biological processes, cellular components, and molecular functions, respectively. These terms included positive regulation of transcription from the RNA polymerase II promoter, response to hypoxia, and inflammatory response. Figure 3B presents the results of the KEGG pathway analysis, revealing 174 pathways associated with common targets. The top 30 pathways were ranked based on significant enrichment and gene count, with the NLRP3 inflammasome signaling pathway, TNF signaling pathway, and pathways in cancer identified as the most prominent. Notably, the NLRP3 inflammasome signaling pathway exhibited lower *p*-values and higher gene counts compared to other pathways, suggesting its potential as a primary mechanism of action for PHCE in treating *NE*.

### 2.4. Molecular Docking

**Figure 4:**
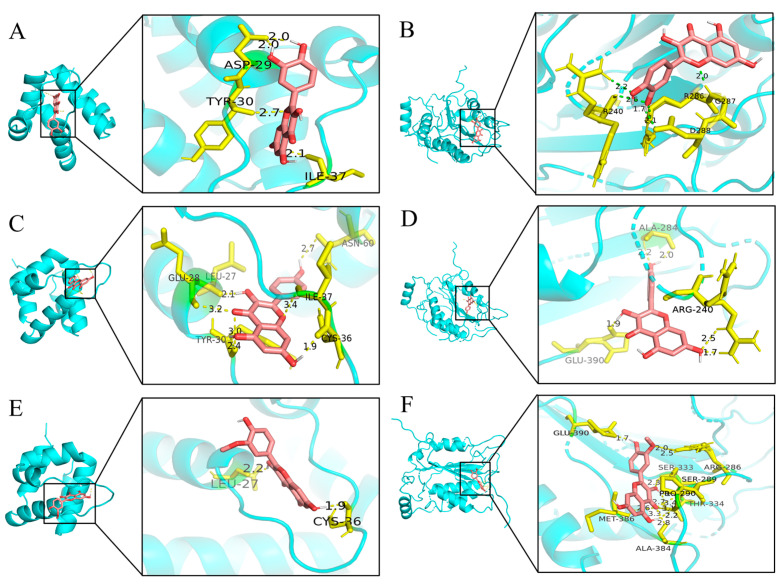
Molecular docking results of main chemical components of PHCE. Quercetin-NLRP3 (**A**), Quercetin-CASP1 (**B**), Kaempferol-NLRP3 (**C**), Kaempferol-CASP1 (**D**), Isorhamnetin-NLRP3 (**E**) and Isorhamnetin-CASP1 (**F**).

Two primary targets, NLRP3 and CASP1, exhibiting the highest degree values within the network diagram, were selected, along with the top components, for molecular docking analysis. The absolute values of docking scores reflect the affinity and conformational stability of component–target interactions. A score exceeding 4.25 indicates a certain binding activity, while values greater than 5.0 and 7.0 suggest good and strong binding activities, respectively [24]. As shown in Table 2, all docking results were negative, and the absolute binding energies of NLRP3 and CASP1 to the ligand surpassed 5.0, indicating favorable ligand–receptor binding activity. Figure 4 illustrates the specific binding site of each protein and the calculated specific binding energy for each target, with those exhibiting superior binding energies highlighted.

### 2.5. MIC and MBC of PHCE

As presented in Table 3, the MIC of PHCE was 3.13 mg/mL. For individual components, patchouli exhibited an MIC of 12.5 mg/mL, *Polygonum hydropiper* L. 6.25 mg/mL, astragalus and licorice 50 mg/mL each, and pulsatilla and Ligustrum lucidum 6.25 mg/mL each. Following PHCE treatment, the bacterial solution was uniformly spread on a sterile TSC solid medium. A colony count below five indicated the MBC, which was found to be 12.5 mg/mL for PHCE, as shown in Table 4. Comparative analysis of PHCE and its constituent fractions revealed that PHCE demonstrated superior bacteriostatic efficacy against *C. Perf*.

### 2.6. Antibacterial Activity Curve of PHCE against C. perf

**Figure 5:**
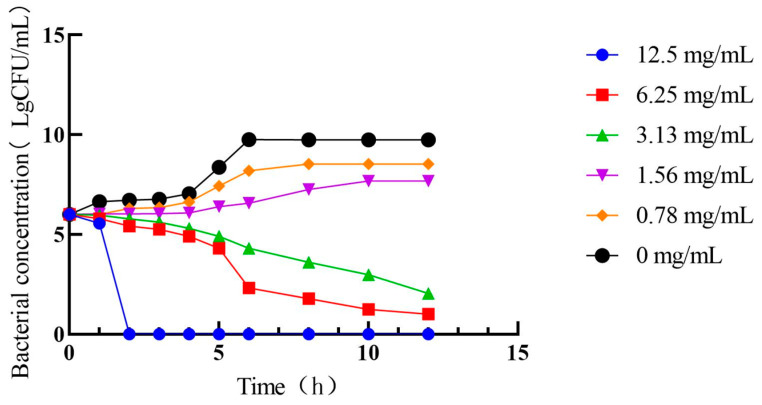
Antibacterial activity curve of PHCE against *C. perf*.

As shown in Figure 5, different PHCE concentrations induced varying growth curve alterations in *C. perf*. At 1/4× MIC (0.78 mg/mL) and 1/2× MIC (1.56 mg/mL) PHCE concentrations, *C. perf* bacterial concentration declined. While growth rates slowed at 1 and 4 h, respectively, bacteria eventually entered a stable phase following rapid logarithmic growth, with final growth approximating that of the 0 mg/mL PHCE control group. Conversely, at MIC and 2× MIC PHCE concentrations, *C. perf* exhibited no logarithmic growth phase, and colony numbers decreased over time, falling below the initial colony count.

### 2.7. Levels of SOD, H_2_O_2_, and MDA in Jejunal and Ileal Tissues

**Figure 6:**
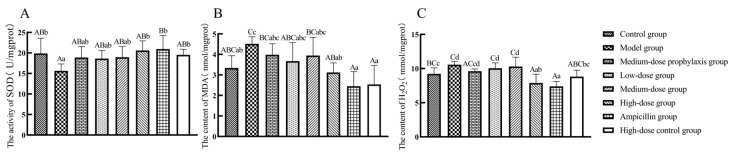
Levels of oxidative stress-related factors in the jejunum: SOD (**A**), MDA (**B**), and H_2_O_2_ (**C**). Data represent mean ± standard error of the mean (*n* = 5). A total of 200 one-day-old healthy broilers were randomly assigned to eight groups, with five replicates per treatment group and five broilers per replicate. The experiment lasted 17 days. Bars labeled with different uppercase letters indicate highly significant differences (*p* < 0.01), while bars labeled with different lowercase letters indicate significant differences (*p* < 0.05). Bars labeled with the same letter indicate no significant difference (*p* > 0.05).

As shown in Figure 6, compared to the control group, SOD enzyme activity in jejunal tissues of model group broilers was significantly lower (*p* < 0.05), while MDA and H_2_O_2_ levels were significantly higher (*p* < 0.05). H_2_O_2_ levels were significantly elevated in jejunal tissues of both the low-dose and medium-dose group broilers (*p* < 0.05), with no significant changes in SOD enzyme activity or MDA levels (*p* > 0.05). The levels of H_2_O_2_ in the jejunal tissues of broilers in both the high-dose group and ampicillin group were significantly lower than those in the control group (*p* < 0.01). There were no significant differences in SOD enzyme activity or MDA levels in the high-dose, high-dose control, or ampicillin groups (*p* > 0.05). Compared to the model group, SOD enzyme activity was significantly or highly significantly increased (*p* < 0.01 or *p* < 0.05) in jejunal tissues of high-dose, high-dose control, and positive control group broilers, accompanied by significantly or highly significantly decreased MDA and H_2_O_2_ levels (*p* < 0.01 or *p* < 0.05). There was no significant change in SOD enzyme activity or MDA level in the low-dose, medium-dose, or medium-dose prophylaxis groups (*p* > 0.05).

**Figure 7:**
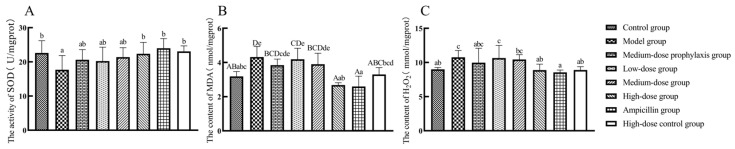
Levels of oxidative stress-related factors in the ileum: SOD (**A**), MDA (**B**), and H_2_O_2_ (**C**). Data represent mean ± standard error of the mean (*n* = 5). A total of 200 one-day-old healthy broilers were randomly assigned to eight groups, with five replicates per treatment group and five broilers per replicate. The experiment lasted 17 days. Bars labeled with different uppercase letters indicate highly significant differences (*p* < 0.01), while bars labeled with different lowercase letters indicate significant differences (*p* < 0.05). Bars labeled with the same letter indicate no significant difference (*p* > 0.05).

Compared to the control group, the model group exhibited significantly decreased SOD activity (*p* < 0.05) and significantly or highly significantly increased MDA and H_2_O_2_ levels (*p* < 0.01 or *p* < 0.05). No significant changes in SOD activity were observed in the ileal tissues of low-dose and medium-dose groups, while MDA and H_2_O_2_ levels were significantly or highly significantly elevated in the medium-dose group (*p* < 0.01 or *p* < 0.05). Relative to the model group, the high-dose, high-dose control, and ampicillin groups displayed significantly or highly significantly increased SOD activity and decreased MDA and H_2_O_2_ levels (*p* < 0.01 or *p* < 0.05). No significant alterations in SOD activity, MDA, or H_2_O_2_ levels were observed in the ileal tissues of low-dose, medium-dose, or medium-dose prevention groups (*p* > 0.05) (Figure 7).

### 2.8. Levels of TNF-α, IL-1β, and IL-6 in Jejunal and Ileal Tissues

**Figure 8:**
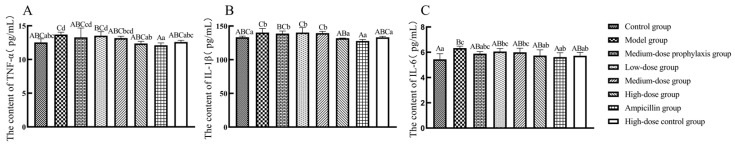
Secretion level of inflammatory factors in jejunum. TNF-α (**A**), IL-1β (**B**), and IL-6 (**C**). Data represent mean ± standard error of the mean (*n* = 5). A total of 200 one-day-old healthy broilers were randomly assigned to eight groups, with five replicates per treatment group and five broilers per replicate. The experiment lasted 17 days. Bars labeled with different uppercase letters indicate highly significant differences (*p* < 0.01), while bars labeled with different lowercase letters indicate significant differences (*p* < 0.05). Bars labeled with the same letter indicate no significant difference (*p* > 0.05).

As shown in Figure 8, compared to the control group, TNF-α secretion levels in jejunal tissues were significantly elevated in model and low-dose group broilers (*p* < 0.05), while IL-1β and IL-6 secretion levels were significantly or highly significantly increased in model, low-dose, and medium-dose group broilers (*p* < 0.01 or *p* < 0.05). IL-1β secretion levels were significantly increased in jejunal tissues of medium-dose prevention group broilers (*p* < 0.05). No significant changes in TNF-α, IL-1β, or IL-6 secretion levels were observed in jejunal tissues of high-dose, high-dose control, or ampicillin groups (*p* > 0.05). Compared to the model group, TNF-α, IL-1β, and IL-6 secretion levels were significantly or extremely significantly reduced in jejunal tissues of high-dose, high-dose control, and ampicillin group broilers (*p* < 0.01 or *p* < 0.05). No significant changes in TNF-α, IL-1β, or IL-6 secretion levels were observed in jejunal tissues of low-dose, medium-dose, or medium-dose prevention groups (*p* > 0.05).

**Figure 9:**
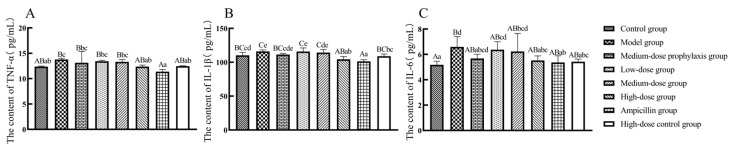
Secretion level of inflammatory factors in ileum. TNF-α (**A**), IL-1β (**B**), and IL-6 (**C**). Data represent mean ± standard error of the mean (*n* = 5). A total of 200 one-day-old healthy broilers were randomly assigned to eight groups, with five replicates per treatment group and five broilers per replicate. The experiment lasted 17 days. Bars labeled with different uppercase letters indicate highly significant differences (*p* < 0.01), while bars labeled with different lowercase letters indicate significant differences (*p* < 0.05). Bars labeled with the same letter indicate no significant difference (*p* > 0.05).

As shown in Figure 9, compared to the control group, ileal tissue TNF-α, IL-1β, and IL-6 secretion levels were significantly or highly significantly elevated (*p* < 0.01 or *p* < 0.05) in model group broilers. Low-dose and medium-dose group broilers exhibited significantly increased IL-6 levels (*p* < 0.05) in ileal tissues. The high-dose and ampicillin groups displayed significantly or highly significantly reduced IL-1β secretion (*p* < 0.01 or *p* < 0.05) in ileal tissues, with no significant changes in TNF-α or IL-6 levels (*p* > 0.05). Compared to the model group, ileal tissue TNF-α, IL-1β, and IL-6 secretion levels were significantly or highly significantly decreased (*p* < 0.01 or *p* < 0.05) in high-dose, high-dose control, and ampicillin group broilers. No significant changes in TNF-α, IL-1β, or IL-6 secretion levels were observed in ileal tissues of low-dose, medium-dose, or medium-dose prevention group broilers (*p* > 0.05).

### 2.9. Injury Scoring of Jejunum and Ileum

Compared to the control group, jejunal and ileal tissue damage scores were significantly higher (*p* < 0.01) in the model and low-dose groups, while no significant differences were observed in the medium-dose prophylaxis, medium-dose, high-dose, high-dose control, and ampicillin groups (*p* > 0.05). Relative to the model group, no significant changes were detected in the medium-dose prophylaxis, low-dose group, and medium-dose groups (*p* > 0.05), whereas jejunal and ileal tissue damage scores were significantly or highly significantly lower (*p* < 0.01 or *p* < 0.05) in the high-dose, high-dose control, and ampicillin groups (Table 5).

### 2.10. Histopathologic Lesions of the Jejunum and Ileum

**Figure 10:**
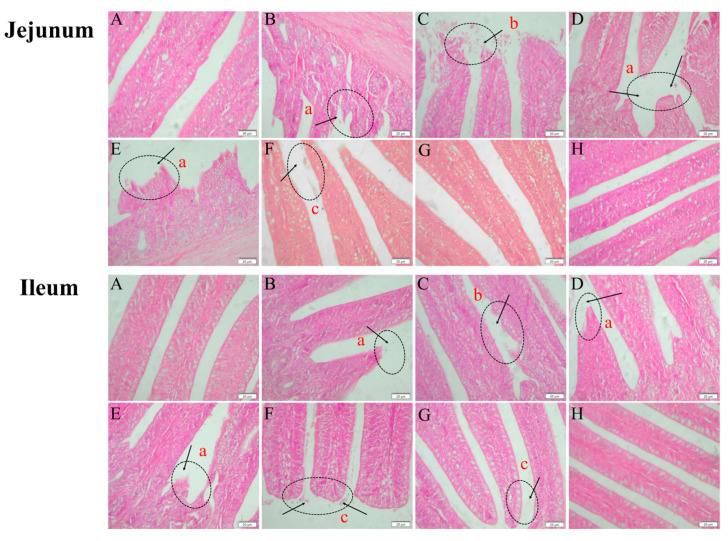
Histopathological changes in the jejunum and ileum of broilers (400× magnification). Control group (**A**), model group (**B**), medium-dose prophylactic group (**C**), low-dose group (**D**), medium-dose group (**E**), high-dose group (**F**), ampicillin group (**G**), and high-dose control group (**H**). Histopathological abnormalities include intestinal villi rupture (a), extensive necrotic shedding of intestinal villi (b), and minor shedding at the tips of intestinal villi (c).

As shown in Figure 10, enterochromaffin structures in the jejunum and ileum of broilers from the model, low-dose, medium-dose, and medium-dose prophylactic groups exhibited breakage with necrotic shedding or disintegration. In contrast, enterochromaffin cells in the jejunum and ileum of the high-dose and ampicillin groups displayed relatively intact structures with only minor detachment at the cellular ends. The jejunal and ileal intestinal villi of broilers in the high-dose control and control groups maintained structural integrity without apparent pathological damage.

### 2.11. The mRNA Levels of NLRP3 Inflammasome Signaling Pathway-Related Factors in Jejunal and Ileal Tissues

**Figure 11:**
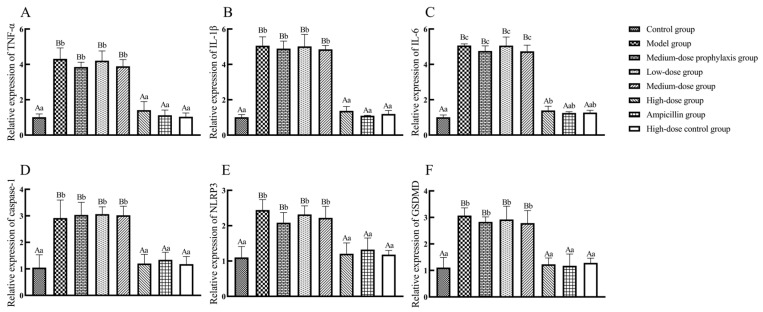
The expression of inflammasome signal pathway-related factors in the jejunum. TNF-α (**A**), IL-1β (**B**), IL-6 (**C**), caspase-1 (**D**), NLRP3 (**E**), and GSDMD (**F**). Data represent mean ± standard error of the mean (n = 5). A total of 200 one-day-old healthy broilers were randomly assigned to eight groups, with five replicates per treatment group and five broilers per replicate. The experiment lasted 17 days. Bars labeled with different uppercase letters indicate highly significant differences (*p* < 0.01), while bars labeled with different lowercase letters indicate significant differences (*p* < 0.05). Bars labeled with the same letter indicate no significant difference (*p* > 0.05).

As shown in Figure 11, mRNA expression levels of TNF-α, IL-1β, IL-6, caspase-1, NLRP3, and GSDMD were significantly elevated (*p* < 0.01) in jejunal tissues of broilers from the model, low-dose, medium-dose, and medium-dose prophylactic groups compared to the control group. In contrast, mRNA expression levels of these same cytokines remained unchanged in jejunal tissues of broilers from the high-dose, high-dose control, and ampicillin groups (*p* > 0.05). Compared to the model group, mRNA expression levels of TNF-α, IL-1β, IL-6, caspase-1, NLRP3, and GSDMD were significantly reduced (*p* < 0.01) in jejunal tissues of broilers from the high-dose, high-dose control, and ampicillin groups. No significant changes in mRNA expression levels of these cytokines were observed in jejunal tissues of broilers from the low-dose or medium-dose prevention groups (*p* > 0.05).

**Figure 12:**
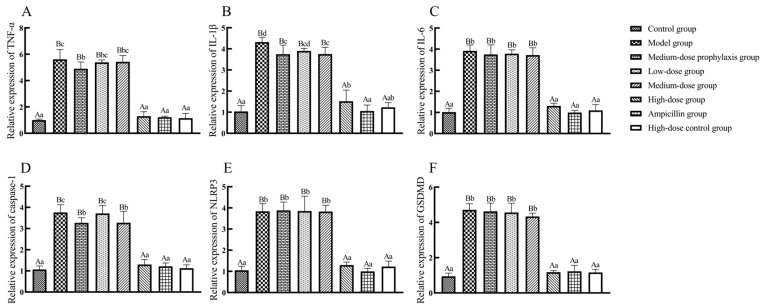
The expression of inflammasome signal pathway-related factors in ileum. TNF-α (**A**), IL-1β (**B**), IL-6 (**C**), caspase-1 (**D**), NLRP3 (**E**), and GSDMD (**F**). Data represent mean ± standard error of the mean (n = 5). A total of 200 one-day-old healthy broilers were randomly assigned to eight groups, with five replicates per treatment group and five broilers per replicate. The experiment lasted 17 days. Bars labeled with different uppercase letters indicate highly significant differences (*p* < 0.01), while bars labeled with different lowercase letters indicate significant differences (*p* < 0.05). Bars labeled with the same letter indicate no significant difference (*p* > 0.05).

As shown in Figure 12, ileal tissue mRNA expression levels of TNF-α, IL-1β, IL-6, caspase-1, NLRP3, and GSDMD were significantly elevated in broilers from the model, low-dose, medium-dose, and medium-dose prophylaxis groups compared to the control group (*p* < 0.01). While IL-6 mRNA levels were significantly increased in the high-dose group (*p* < 0.05), TNF-α, IL-1β, caspase-1, NLRP3, and GSDMD mRNA levels remained unchanged in the high-dose, high-dose drug control, and ampicillin groups (*p* > 0.05). Compared to the model group, ileal tissue mRNA expression levels of TNF-α, IL-1β, IL-6, caspase-1, NLRP3, and GSDMD were significantly reduced in the high-dose, high-dose control, and ampicillin groups (*p* < 0.01). No significant changes in mRNA expression levels of these cytokines were observed in the low-dose, medium-dose, or medium-dose prophylaxis groups (*p* > 0.05).

## 3. Discussion

PHCE, formulated based on traditional Chinese veterinary principles, comprises six Chinese herbs including *Polygonum hydropiper* L., *Patchouli, Pulsatilla*, *Astragalus*, *Ligustrum lucidum*, and *Licorice*, with *Polygonum hydropiper L.* serving as the primary therapeutic agent [14]. Primarily affecting broiler chicks aged 2–6 weeks, *NE* is induced by *C. perf* and represents a significant bacterial intestinal disease in poultry [25]. Analysis of the PHCE herb–compound–target network identified flavonoids, such as quercetin, kaempferol, and isorhamnetin, as primary active components. Activation of the NLRP3 inflammasome stimulates caspase-1, leading to the release of inflammatory cytokines IL-1β and IL-18 and subsequent cellular pyroptosis. However, NLRP3 inflammasome hyperactivation can induce pathological inflammation [26]. Studies have demonstrated that flavonoids, including quercetin, kaempferol, and isorhamnetin, effectively inhibit NLRP3 inflammasome activation, ameliorating various inflammatory conditions by targeting the NLRP3 inflammasome and suppressing its mediated inflammatory responses [27,28,29]. Interestingly, PPI network analysis, GO analysis, and KEGG pathway enrichment analysis identified NLRP3 and CASP1 as key PHCE targets against *NE*, suggesting the NLRP3 inflammasome pathway as a potential mechanism of action. Molecular docking analysis revealed strong binding affinities between PHCE’s primary active components (quercetin, kaempferol, and isorhamnetin) and the target proteins NLRP3 and CASP1, indicating that PHCE may counteract *NE* by inhibiting NLRP3 inflammasome activation.

Herbal medicines, with their antimicrobial, antioxidant, and anti-inflammatory properties, and their reduced propensity for bacterial resistance, are promising alternatives to antibiotics in preventing and treating bacterial diseases [30]. PHCE, composed of *Polygonum hydropiper* L., *Patchouli, Pulsatilla*, *Astragalus*, *Ligustrum lucidum*, and *Licorice*, aligns with traditional Chinese veterinary medicine, with *Polygonum hydropiper* L. serving as the primary therapeutic agent. Zhou et al. [31] reported the potent bacteriostatic effects of *hydropiper* L. decoction against *Salmonella dysentery*, *Escherichia coli*, and *Staphylococcus* aureus (MICs of 15.62 g/L), as well as *Candida albicans* and *Pseudomonas aeruginosa* (MICs of 31.25 g/L). Zhu et al. [32] employed a compound preparation containing *Polygonum hydropiper* L., *Sapium sebiferum* leaves, and neem to treat red crucian carp fingerling disease, achieving a 100% worm kill rate within 24 h at a dose of 8 g/L. These studies collectively suggest that PHCE or compound formulations centered around *Polygonum hydropiper* L. possess significant antibacterial potential and hold promise for preventing and treating animal diseases. The current study determined MIC and MBC values of 3.13 mg/mL and 12.5 mg/mL, respectively, against *C. perf* type A, confirming PHCE’s potent antibacterial activity. However, given the potential discrepancies between in vitro and in vivo antimicrobial activities of traditional Chinese medicines or their compounds, further in vivo investigations are necessary to evaluate PHCE’s efficacy against *C. perf*-induced *NE*.

Network pharmacology analysis revealed that most PHCE active ingredients are flavonoids, possessing antioxidant properties through antioxidant enzyme activation and reduced oxygen-containing free radical levels. SOD, MDA, and H_2_O_2_ play crucial roles in maintaining redox balance in animals. SOD, an antioxidant enzyme, catalyzes the disproportionation of superoxide anions into H_2_O_2_ and molecular oxygen. H_2_O_2_, in the presence of iron chelates, reacts with oxygen to generate harmful hydroxyl radicals. MDA exhibits cytotoxicity, while the antioxidant enzyme CAT scavenges H_2_O_2_ [33]. Flavonoids exert antioxidant effects by increasing antioxidant enzyme activity and chelating metal ions [34]. Wang et al. [35] demonstrated that *Angelica dahurica* (polymethoxylated flavonoids) attenuated oxidative stress damage in astrocytes by reducing ROS and MDA production in a hypoxia-induced oxidative stress model. Tao et al. [36] reported that a compound containing *Polygonum hydropiper* L. and astragalus enhanced broiler chicken antioxidant capacity by increasing SOD enzyme activity when added to the basal diet. In this in vivo experiment, high-dose and ampicillin groups exhibited significantly or highly significantly increased SOD enzyme activity and decreased MDA and H_2_O_2_ levels in jejunal and ileal tissues compared to the model group, aligning with previous findings. While the ampicillin group demonstrated a stronger effect on increasing SOD enzyme activity and reducing MDA and H_2_O_2_ levels in broiler and ileal tissues compared to the medium- and low-dose groups, the high-dose group exhibited comparable efficacy. These results suggest that high-dose PHCE can alleviate oxidative stress damage induced by *C. perf NE* by enhancing antioxidant capacity.

Oxidative stress and inflammation can contribute to intestinal injury in poultry, with a reciprocal relationship between the two processes. Inflammatory cells release substantial quantities of reactive substances at inflammation sites, exacerbating oxidative stress. Conversely, reactive oxygen and nitrogen species (ROS/RNS) can initiate intracellular signaling cascades and upregulate pro-inflammatory gene expression [37,38,39,40]. Flavonoids possess diverse biological activities, including antioxidant and anti-inflammatory properties. *Angelica dahurica* attenuated hepatic injury in a mouse model of hepatic ischemia by enhancing antioxidant capacity and reducing TNF-α, IL-1β, and IL-6 mRNA expression [41]. Similarly, compound formulations containing *Polygonum hydropiper* L. have demonstrated comparable effects. Yang et al. [42] reported that *Polygonum hydropiper* L.-based Changyanning compound ameliorated ulcerative colitis in rats by increasing antioxidant enzyme activity and reducing MDA and TNF-α levels. In the current in vivo experiment, the high-dose and ampicillin groups exhibited the most pronounced effects, significantly or markedly reducing TNF-α, IL-1β, and IL-6 secretion and mRNA expression in the jejunum and ileum of broilers compared to the model group.

Inflammatory cytokines contribute to host defense against bacterial and other microbial invasions, but their overexpression disrupts biological homeostasis, triggering an inflammatory cascade that compromises intestinal integrity and barrier function [43,44]. Conversely, these cytokines protect the intestinal tract from pathogen-induced damage. Liu et al. [18] demonstrated that licorice chalcone A reduced intestinal damage scores and preserved intestinal villus structure with minimal pathological changes in *C. perf*-induced *NE* in broiler chickens. In the present study, all treatment groups (low-dose, medium-dose, high-dose, medium-dose prophylaxis, high-dose control, and ampicillin control) reduced intestinal damage scores. However, only the high-dose, high-dose control, and ampicillin groups exhibited relatively intact intestinal villi structures in jejunal and ileal histological sections, with minimal necrotic detachment at intestinal villus apices in the high-dose group. Pathological damage in the jejunum and ileum of ampicillin-treated broilers was less severe than in the low-dose and medium-dose groups, resembling that of the control group. The high-dose and high-dose control groups displayed similar levels of jejunal and ileal pathological damage as the ampicillin group. The high-dose control group, lacking antibacterial treatment and receiving only high-dose PHCE, exhibited effects on antioxidant capacity, inflammatory cytokine secretion and expression, and intestinal damage similar to the control group, indicating the safety and non-toxicity of high-dose PHCE in broilers. Moreover, these findings suggest that high-dose PHCE effectively treats *C. perf*-induced *NE*.

Inflammation is a biological process safeguarding the body from pathogen invasion and cellular stress signals. The inflammatory response is triggered by inflammasome activation (intracellular protein complexes). Stress-induced danger signals encompass various molecules, including cellular debris (e.g., DNA fragments, ATP), pathogen-associated molecular patterns (e.g., bacterial LPS, viral dsRNA), and cytokines/chemokines released by damaged or stressed cells [45]. These molecules are recognized by the immune system and cellular receptors, activating inflammasomes and initiating the inflammatory response. PAMPs and DAMPs stimulate NLRP3 inflammasome activation, which subsequently activates caspase-1. Caspase-1 cleaves pro-IL-1β and pro-IL-18 into active IL-1β and IL-18, releasing them extracellularly. Additionally, caspase-1 cleaves GSDMD, a member of the gasdermin family, resulting in cell perforation and pyroptosis. However, excessive inflammasome activation can induce pathological inflammation [26]. Studies have demonstrated the anti-inflammatory effects of flavonoids through inflammasome inhibition. For instance, Tsai et al. [46] observed that the flavonoid gallocatechin gallate (EGCG) attenuated lupus nephritis in mice by inhibiting caspase-1 activation through decreased NLRP3 mRNA expression, reducing IL-1β and IL-18 production. Jiang et al. [47] investigated the effects of quercetin on mice with sodium urate crystal-induced gouty arthritis, finding that quercetin ameliorated symptoms by inhibiting NLRP3, caspase-1, and IL-1β mRNA expression in knee joints. ROS, as upstream regulators of NLRP3 inflammasome activation, are inhibited by flavonoids, which also reduce MDA production [48]. For example, lidocaine suppresses ROS production, inhibiting NLRP3 inflammasome activation and preventing THP-1 cell pyroptosis [49]. Apigenin and fusaricin decrease MDA levels in Adriamycin-induced renal injury and glyoxylate-induced renal tissue injury, respectively [50,51]. H_2_O_2_ and ∙OH, both ROS, contribute to MDA formation through lipid oxidation [52]. Compared to the model group, the high-dose PHCE group exhibited significantly or highly significantly lower H_2_O_2_ and MDA levels in jejunal and ileal tissues, suggesting reduced ROS production. Concurrently, NLRP3, caspase-1, IL-1β, and GSDMD mRNA expression was significantly or highly significantly elevated in jejunal and ileal tissues of high-dose, high-dose control, and ampicillin groups, but not in low-dose, medium-dose, or medium-dose prophylaxis groups. The high-dose control group (no antibacterial treatment) and ampicillin group (antibiotic effective against *C. perf*) served as controls. These findings suggest that high-dose PHCE protects against *C. perf*-induced NE by inhibiting NLRP3 inflammasome activation through reduced ROS production, subsequently attenuating intestinal damage.

## 4. Materials and Methods

### 4.1. Bacterial Strains and Drugs

*C. perf* type A (ATCC13124) was purchased from the American type culture collection (ATCC, Rockville, MD, USA), and anaerobically cultured in Brain heart infusion (BHI) broth (Qingdao Hopebio Co., Qingdao, China) or on Tryptose sulfite–cyloserine (TSC) agar base (Qingdao Hopebio Co. Qingdao, China) at 37 °C. The raw materials for the test drug, such as *Polygonum hydropiper L.*, *Patchouli*, *Pulsatilla*, *Astragalus*, *Ligustrum lucidum*, and *Licorice*, were purchased from Gaoxiong Chinese Medicine (Kaohsiung, China).

### 4.2. Screening the Active Ingredients and Targets of PHCE

The compounds and the corresponding targets of *Polygonum hydropiper* L., *Patchouli*, *Pulsatilla*, *Astragalus*, *Ligustrum lucidum*, and *Licorice* in PHCE were obtained from the databases TCMSP (https://old.tcmsp-e.com/tcmsp.php, accessed on 15 June 2024) and CNN (https://www.cnki.net/, accessed on 15 June 2024). We retrieved all components of the PHCE compounds from TCMSP and CNN, which had oral bioavailability (OB) ≥ 30% and drug-likeness (DL) ≥ 0.18 as screening conditions to retrieve the active components and targets of action of PHCE [53,54].

### 4.3. NE Target Acquisition

To identify potential therapeutic targets for *NE*, the GeneCards database (https://www.genecards.org/, accessed on 16 June 2024) and PharmGKB database (https://www.pharmgkb.org/, accessed on 16 June 2024) were queried using the keyword “Necrotic Enteritis”. Extracted disease targets from both databases were then merged and subjected to de-weighting to account for potential redundancies. Subsequently, the de-weighted list of potential targets was imported into the UniPort database (https://www.uniprot.org/, accessed on 16 June 2024) for name standardization to ensure a consistent nomenclature for NE targets.

### 4.4. Construction of Active Compound–Target Networks

Cytoscape is a network biology visualization and analysis software that enables the visualization of molecular interactions and biological processes [55]. The PHCE chemical composition and target files were imported into Cytoscape 3.9.1 to construct a compound–target network. In this network, each compound or target is represented by a node, and the relationships between them are depicted as connecting lines.

### 4.5. Construction of PPI Network

To identify potential PHCE targets for treating *NE*, the active ingredients of PHCE and known *NE* targets were imported into a bioinformatics platform (https://www.bioinformatics.com.cn/, accessed on 19 June 2024) to generate a Venn diagram. Overlapping genes within the diagram were considered potential targets for PHCE action against *NE*. These intersecting genes were subsequently uploaded to the STRING database (https://cn.string-db.org/, accessed on 20 June 2024) with a high confidence interaction score threshold of 0.9 [56]. Irrelevant nodes were hidden, and default settings were maintained for other parameters. The resulting interaction network data were exported in “.tsv” format and imported into Cytoscape 3.9.1 software. Network node properties, including Degree, Betweenness Centrality, and Closeness Centrality, were calculated within Cytoscape [57]. Nodes exceeding the median values for these properties were identified as key targets and used to construct the final PPI network.

### 4.6. Functional Enrichment Analysis

Genes identified at the intersection of PHCE’s mechanism of action and *NE* were imported into the DAVID database (https://david.ncifcrf.gov/summary.jsp, accessed on 21 June 2024) for GO analysis with background enrichment against the KEGG pathway database [58]. The top 10 significantly enriched (*p*-value < 0.01) GO terms in each category (biological process, cellular component, and molecular function) were selected, along with the top 30 pathways ranked by gene enrichment [59]. Subsequently, GO and KEGG data were uploaded to the microbiometrics platform for visual analysis.

### 4.7. Molecular Docking Studies

Leveraging network pharmacology, the top three key components of PHCE effective against *NE* were identified and subjected to molecular docking simulations with the two most promising target proteins from the PPI network. Here, the key components act as ligands, and the target proteins function as receptors. The 3D structures of the target proteins were downloaded in PDB format from the RCSB PDB database (http://www.rcsb.org/, accessed on 22 June 2024) while ligand structures were retrieved in SDF format from the TCMSP and PubChem databases (https://pubchem.ncbi.nlm.nih.gov/, accessed on 23 June 2024). Open Babel 2.4.1 was used to convert ligand SDF files into MOL2 format, which is suitable for docking simulations. Finally, PyMoL 5.2.7 and AutoDock Vina [60] were employed to perform the docking simulations between the key PHCE ingredients and core target proteins. The minimum binding efficiency served as the metric for assessing ligand–receptor binding activity.

### 4.8. Preparation of PHCE

PHCE was prepared using a hydrodecoction–alcohol method. A 20 g mixture of six herbs (*Polygonum hydropiper* L., *Patchouli*, *Pulsatilla*, *Astragalus*, *Ligustrum lucidum*, and *Licorice*), sieved through a 100-mesh sieve, was decocted three times in ten volumes of distilled water at 100 °C for 1.5 h each. The combined filtrates (passed through 8-layer gauze) were centrifuged at 3000 rpm for 30 min, and the supernatant was concentrated using a rotary evaporator at 60 °C. Subsequently, 95% ethanol was added to achieve an 80% ethanol concentration, and the solution was left to stand for 24 h. After filtration through eight layers of gauze and centrifuging at 3000 rpm for 30 min, the ethanol was recovered using a rotary evaporator, and the solid obtained (20 g) was suspended in the corresponding medium to have 20 mL of solution (conc. 1 g/mL). The prepared PHCE was stored at 4 °C for subsequent use.

### 4.9. Determination of MIC and MBC

PHCE, *Polygonum hydropiper* L., patchouli, pulsatilla, astragalus, *Ligustrum lucidum*, and licorice medicinal liquids were autoclaved at 100 °C for 30 min for sterilization. Subsequently, 4 mL of each drug solution was pipetted into 1 mL of BHI medium, yielding an 800 mg/mL concentration. Serial two-fold dilutions were performed using BHI liquid medium to prepare 10 drug concentrations ranging from 0.78 mg/mL to 400 mg/mL. The *C. perf* bacterial solution in the logarithmic growth phase was diluted to about 2 × 10^6^ CFU/mL in BHI medium. A sterile 96-well plate was then used to introduce 100 μL of the bacterial solution to each well, followed by the addition of 100 μL of the prepared drug solutions at varying concentrations, resulting in final drug concentrations ranging from 0.39 mg/mL to 200 mg/mL and a final bacterial concentration of 1 × 10^6^ CFU/mL. Positive (100 μL BHI medium + 100 μL bacterial solution) and negative (200 μL BHI medium) controls were established in triplicate. In 96-well plates, each experimental group was repeated four times, and three 96-well plates were used for 12 replicates. All 96-well plates were incubated for 12–18 h at 37 °C in a 2.5 L round-bottomed vertical anaerobic culture bag (Qingdao Hopebio Co.) containing a 2.5 L anaerobic gas-generating bag and oxygen indicator. Following incubation, the cultures were mixed, and the OD600 nm value was measured using an enzyme-labeling instrument. An absorbance change of less than 0.05 indicated effective bacterial growth inhibition, defining the MIC. An absorbance change of less than 0.05 at OD600 nm was considered the absence of bacterial growth, allowing for the determination of the MIC of PHCE, *Polygonum hydropiper* L., patchouli, pulsatilla, astragalus, *Ligustrum lucidum*, and licorice medicinal liquids against *C. perf* based on the measured absorbance values.

To determine the MBC of PHCE against *C. perf*, five PHCE drug concentrations (0.78 mg/mL, 1.56 mg/mL, 3.13 mg/mL, 6.25 mg/mL, and 12.5 mg/mL) were prepared. One hundred microliters of bacterial suspension were evenly inoculated onto a sterile TSC solid medium and placed within a 2.5 L round-bottom vertical anaerobic culture bag. A 2.5 L anaerobic gas production package and oxygen indicator were added before incubation at 37 °C overnight. The PHCE concentration resulting in fewer than five colonies on the medium was defined as the MBC against *C. perf*.

### 4.10. Determination of Antimicrobial Activity

*C. perf* liquid was diluted to 1 × 10^6^ CFU/mL in BHI medium and distributed into tubes. PHCE was introduced at final concentrations of 0 mg/mL, 0.78 mg/mL, 1.56 mg/mL, 3.13 mg/mL, 6.25 mg/mL, and 12.5 mg/mL, respectively. Tubes were incubated statically at 37 °C, and 100 μL bacterial aliquots were collected at 0, 1, 2, 3, 4, 6, 8, 10, and 12 h for bacterial count determination via plaque assay, with triplicate samples at each time point. Time–kill curves were generated, plotting time (hours) on the horizontal axis and colony number (log) on the vertical axis.

### 4.11. Animals and Treatment

Two hundred one-day-old healthy yellow-feathered broilers were purchased from Guangxi Shengde Poultry; the cages and equipment used in the test were thoroughly disinfected, and the coops were fumigated. Broilers were routinely vaccinated, fed, and watered ad libitum, and the coops were cleaned and disinfected regularly. Environmental temperature control was as follows: 32–34 °C in the first week, the temperature was reduced by 2–3 °C every week, and the humidity of the chicken house was controlled at 50–60%. The basal feed (BF) was the same corn–soybean meal type, formulated with reference to the Chicken Feeding Standard (2004), and its composition and nutrient content are shown in Table 6 [61]. The present experiment was carried out at the hatchery facility of the Laboratory of Histoembryology, College of Animal Science and Technology of Guangxi University, and the broiler rearing facility of Guangxi Key Laboratory of Animal Breeding, Disease Control and Prevention, Nan-Ning City, China. All animal procedures were approved by the Animal Care and Welfare Committee of Guangxi University, China (Protocol GXU-2020-008).

Two hundred one-day-old healthy broilers were randomly assigned to eight groups: control, model, medium-dose prophylaxis, low-, medium-, and high-dose ampicillin, and high-dose drug control. Each treatment group contained five replicates of five broilers. Broilers received a basal diet throughout the experiment (Table 7). On day 17, chicks were slaughtered via CO_2_ asphyxiation. Under aseptic conditions, the abdominal cavity was opened to observe and score jejunal and ileal pathological changes. One-centimeter segments from the mid-sections of the jejunum and ileum were collected, fixed in precooled 4% paraformaldehyde, washed with sterilized phosphate-buffered saline (PBS) to remove intestinal contents, and stored at −80 °C in liquid nitrogen for rapid freezing.

### 4.12. Determination of Oxidation and Antioxidant Indexes

Jejunal and ileal tissues were retrieved from −80 °C storage and minced into small pieces using autoclaved scissors. Approximately 0.1 g of tissue from each group was weighed and homogenized in 0.9 mL PBS containing sterile grinding beads using a high-speed tissue grinder. The homogenate was centrifuged at 5000× *g* for 10 min, and the supernatant was carefully transferred to a new centrifuge tube for subsequent analysis. SOD activity and H_2_O_2_ and MDA levels were quantified according to kit instructions. The H_2_O_2_ (20220509), MDA (20220428), and SOD (20220507) kits were procured from Nanjing Jiancheng Bioengineering Institute (Nanjing, China).

### 4.13. Determination of TNF-α, IL-1β and IL-6

TNF-α, IL-1β, and IL-6 levels were quantified in jejunal and ileal tissue homogenate supernatants using ELISA kits (TNF-α [MM-093801], IL-1β [MM-3691001], and IL-6 [MM-052101]) procured from Jiangsu Enzyme Immunity Industry Co., Ltd. (Taizhou, China), following the manufacturer’s protocol.

### 4.14. Scoring of Jejunal and Ileal Lesions

Ocular pathologic changes were observed within a 5 cm segment of the jejunum and the anterior ileum. Intestinal injury was scored using a 0–6 point scale adapted from Shojadoost et al. [62]: 0 (no apparent injury), 1 (intestinal wall thinning and brittleness), 2 (1–5 necrotic foci), 3 (6–15 necrotic foci), 4 (16 or more necrotic foci), 5 (a 2–3 cm necrotic sheet), and 6 (a large, diffuse necrotic area).

### 4.15. Observation of Histopathologic Changes in the Jejunum and Ileum

Jejunal and ileal tissues were fixed in 4% paraformaldehyde for 24 h, followed by dehydration, clearing, wax embedding, sectioning, and hematoxylin–eosin (HE) staining to produce histopathological sections. Microscopic examination under low magnification assessed the morphology and structure of jejunum and ileum tissues, while high-magnification examination focused on intestinal villi morphology and structure.

### 4.16. RNA Extraction and Gene Expression Analysis

Approximately 50–100 mg of jejunum and ileum tissues, stored at −80 °C, were transferred to sterile, enzyme-free microcentrifuge tubes. Sterile, enzyme-free grinding beads were added, followed by 1 mL of RNA isolator reagent. The samples were homogenized using a high-speed tissue grinder, subsequently centrifuged at 12,000× *g* for 5 min at 4 °C, and the supernatant was transferred to a sterile, enzyme-free microcentrifuge tube. Total RNA from intestinal tissues was extracted using the RNA isolator Total RNA Extraction Reagent kit following sample processing.

RNA purity was assessed by spectrophotometric determination of OD260/280 values, while integrity was verified using 1% agarose gel electrophoresis. Reverse transcription employed the All-In-One 5× RT MasterMix kit (Nanjing Ai Bi MnegBiological Material Co., Ltd., Nanjing, China), generating cDNA from 1 μL RNA template, 4 μL MasterMix, and 15 μL ddH_2_O through incubation at 37 °C for 15 min, 60 °C for 10 min, and 95 °C for 3 min. cDNA was stored at −20 °C. RT-qPCR primers targeting GADPH, caspase-1, IL-1β, NLRP3, IL-6, GSDMD, and TNF-α genes were designed using Primer Express 6.0 software based on GenBank sequences and synthesized by Shanghai Sangon Biotech Co., Shanghai, China (Table 8). Real-time PCR reactions were conducted using BlasTaq™ 2× qPCR MasterMix (Nanjing Ai Bi MnegBiological Material Co., Ltd.), comprising 10 μL MasterMix, 0.5 μL of each primer (10 μM), 1 μL cDNA, and 8 μL ddH_2_O. A Light Cycler 96 real-time fluorescence quantitative PCR instrument (Roche, Basel, Switzerland) was used with the following cycling conditions: initial denaturation at 95 °C for 3 min, followed by 40 cycles of denaturation at 95 °C for 15 s and annealing/extension at 60 °C for 60 s.

### 4.17. Statistics Analysis 

SPSS 23 software was used for statistical analysis. One-way analysis of variance (ANOVA) was used to test the main effect. When the differences were significant (*p* < 0.05), the group means were further compared using Tukey’s test. Data were presented as mean ± standard deviation, with significance levels set at *p* < 0.05 and high significance at *p* < 0.01. The analysis for lesion score was performed using the nonparametric Kruskal–Wallis test. Graphical representations were generated using GraphPad Prism 9.

## 5. Conclusions

In summary, network pharmacology and molecular docking analyses indicated that PHCE’s primary active components, including flavonoids such as quercetin, kaempferol, and isorhamnetin, potentially counteract *NE* by modulating the NLRP3 inflammasome signaling pathway. In vitro experiments demonstrated that both PHCE and its constituent herbs exhibit antibacterial properties against *Clostridium perfringens*, with PHCE demonstrating superior efficacy. In vivo studies align with network pharmacology findings, revealing that PHCE (2 g/kg) is safe and effectively mitigates oxidative stress in NE-infected broilers by enhancing intestinal antioxidant capacity. Moreover, PHCE inhibits NLRP3 inflammasome activation, potentially through reduced ROS production, leading to decreased expression of NLRP3 inflammasome signaling pathway-related factors. Consequently, PHCE attenuates jejunal and ileal inflammation, preserving intestinal integrity. The findings of this study provide a theoretical basis for the clinical application of PHCE. The active ingredients and content in PHCE still need further study, and the mechanism of PHCE against *NE* should be verified by knockout and over-expression of genes.

## Figures and Tables

**Table 1:** Top ten compounds information of PHCE network.

Mol ID	Compound	Degree	Closeness Centrality	Betweenness Centrality	OB	DL
MOL000098	quercetin	605	0.51057	0.39401	46.43	0.28
MOL000422	kaempferol	188	0.41936	0.07805	41.88	0.24
MOL000354	isorhamnetin	84	0.39302	0.02112	49.6	0.31
MOL000392	formononetin	71	0.39211	0.03092	69.67	0.21
MOL000358	β-sitosterol	50	0.39579	0.02882	36.91	0.75
MOL000006	luteolin	47	0.41523	0.08765	36.16	0.25
MOL000378	7-*O*-methylisomucronulatol	30	0.40143	0.01798	74.69	0.3
MOL003896	7-Methoxy-2-methyl isoflavone	28	0.39953	0.01247	42.56	0.2
MOL004328	Calycosin	27	0.39302	0.08517	47.75	0.24
MOL000417	naringenin	26	0.38585	0.00097	59.29	0.21

Note: OB (Oral Bioavailability) refers to the rate and extent to which a drug is absorbed into the bloodstream from a dosage form. DL (drug-likeness) indicates the similarity of a compound to known drugs.

**Table 2:** Docking results of core target proteins and core active components.

Active Components	NLRP3	CASP1
quercetin	–5.71	–5.72
kaempferol	–6.28	–5.31
isorhamnetin	–5.79	–6.15

**Table 3:** MIC of Chinese herbs against *C. perf*.

Drug	Drug Concentration (mg/mL)										Negative	Positive
	200	100	50	25	12.5	6.25	3.13	1.56	0.78	0.39		
PHCE	−	−	−	−	−	−	−	+	+	+	−	+
*Patchouli*	−	−	−	−	−	+	+	+	+	+	−	+
*Polygonum hydropiper* L.	−	−	−	−	−	−	+	+	+	+	−	+
*Astragalus*	−	−	−	+	+	+	+	+	+	+	−	+
*Pulsatilla*	−	−	−	−	−	−	+	+	+	+	−	+
*Ligustrum lucidum*	−	−	−	−	−	−	+	+	+	+	−	+
*Licorice*	−	−	−	+	+	+	+	+	+	+	−	+

Note: “+” indicates bacterial growth; “−” indicates no bacterial growth.

**Table 4:** Determination of *C. perf* MBC by PHCE.

	0.78 mg/mL	1.56 mg/mL	3.13 mg/mL	6.25 mg/mL	12.5 mg/mL
*C. perf*	+	+	+	+	−

Note: “+” indicates more colonies; “−” indicates that the number of colonies is less than five.

**Table 5:** Effect of PHCE on jejunal and ileal lesion scores.

Treatments	Rank Score Means	
	Jejunum	Ileum
Control group	5.5 ^Aa^	6.0 ^Aa^
Model group	36.2 ^Bb^	34.8 ^Bb^
Medium dose Prophylaxis group	27.0 ^ABab^	26.2 ^ABab^
Low-dose group	32.1 ^Bb^	32.7 ^Bb^
Medium-dose group	27.4 ^ABab^	27.7 ^ABab^
High-dose group	11.3 ^ABa^	11.8 ^ABa^
Ampicillin group	10.6 ^ABa^	9.4 ^ABa^
High-dose control group	8.9 ^Aa^	9.4 ^ABa^

Note: Data represent rank score means (*n* = 5). Rank score means and difference in rank score means were calculated by the Kruskal–Wallis test. Data with different uppercase letters superscripts differ highly significantly (*p* < 0.01), data with varying letters of lowercase superscripts differ significantly (*p* < 0.05), and data with common superscripts denote no significant difference (*p* > 0.05).

**Table 6:** Basal feed composition and nutritional level (air-dried basis).

Ingredients	Contents (%)	Nutrient Level	Contents (%)
Grain	51.73	Crude protein	21.50
Soybean meal	40.73	Lys	1.17
Soybean oil	3.36	Met	0.59
Calcium phosphate	1.92	Calcium	1.00
Limestone	1.16	Available phosphorus	0.45
Salt	0.35	Met + Cystine	0.90
DL-Met	0.26		
50% Choline chloride	0.25		
Mineral premix	0.20		
Vitamin premix	0.24		
Total	100.00		

Note: (1) Mineral premix provided the following per kg of diet: Mn, 100 mg; Zn, 75 mg; Fe, 80 mg; Cu, 8 mg; I, 0.35 mg; Se, 0.15 mg. (2) Vitamin premix provided the following per kg of diet: VA 12 500 IU, VD_3_ 2500 IU, VE 30 IU, VK 2.65 mg, VB_1_ 2 mg, VB_2_ 6 mg, VB_12_ 0.025 mg, biotin 0.0325 mg, folic acid 1.25 mg, niacin 12 mg, and niacin 50 mg. (3) Metabolism, 3.35 Mcal/kg.

**Table 7:** Experimental group classification by dosage and stimulation protocols.

Group	1–6 d	7–13 d	14–16 d
Control group	BF	BF	PBS
Model group	BF	BF	*C. perf*
Medium-dose Prophylaxis group	BF	BF + 1 g/kg PHCE	*C. perf* + 1 g/kg PHCE
Low-dose group	BF	BF	*C. perf* + 0.5 g/kg PHCE
Medium-dose group	BF	BF	*C. perf* + 1 g/kg PHCE
High-dose group	BF	BF	*C. perf* + 2 g/kg PHCE
Ampicillin group	BF	BF	*C. perf* + 1 g/kg ampicillin
High-dose control group	BF	BF	2 g/kg PHCE

Note: BF: basal feed. Except for the medium-dose prophylaxis group, the other seven groups were fed basal feed from 1 to 13 days. The control group and model group were fed 1 mL sterile PBS and 1 × 10^9^ CFU/mL *C. perf* bacterial solution once a day from 14 to 16 d, respectively, with the same basal feed. The medium-dose prophylaxis group was fed a basal feed from 1 to 6 d, 1 g/kg PHCE was added to the basal feed from 7 to 16 d, and 1 mL 1 × 10^9^ CFU/mL *C. perf* bacterial solution was fed once a day from 14 to 16 d. The high-dose control group was fed a basal feed from 1 to 13 days, and 2 g/kg PHCE was added to the basal feed from 14 to 16 days. The low-dose group, medium-dose group, high-dose group, and ampicillin group were fed basal feed from 1 to 13 days, 1 mL of 1 × 10^9^ CFU/mL *C. perf* bacterial solution from 14 to 16 d, and 0.5 g/kg, 1 g/kg, 2 g/kg PHCE, and 1 g/kg ampicillin were added to the basal diet, respectively, once a day. All groups were sampled at 17 d.

**Table 8:** The sequence of primers.

Gene	Primer Name	Primer Sequence (5′→3′)
*GADPH*	GADPH-F	5′-CTGGCAAAGTCCAAGTGGTG-3′
	GADPH-R	5′-AGCACCACCCTTCAGATGAG-3′
*caspase-1*	caspase-1-F	5′-TTCCTTCAACACCATCTACG-3′
	caspase-1-R	5′-GGTGAGCTTCTCTGGTTTTA-3′
*IL-1β*	IL-1β-F	5′-AGCAGCCTCAGCGAAGAGACC-3′
	IL-1β-R	5′-GTCCACTGTGGTGTGCTCAGAATC-3′
*NLRP3*	NLRP3-F	5′-AGCTACCACACATCTAGGAT-3′
	NLRP3-R	5′-GGTGTCCAAATCCTCAATCT-3′
*IL-6*	IL-6-F	5′-AAGTTCACCGTGTGCGAGAA-3′
	IL-6-R	5′-TCAGGCATTTCTCCTCGTCG-3′
*GSDMD*	GSDMD-F	5′-ACTGAGGTCCACAGCCAAGAGG-3′
	GSDMD-R	5′-GCCACTCGGAATGCCAGGATG-3′
*TNF-α*	TNF-α-F	5′-CTTCCTGCTGGGGTGCATAG-3′
	TNF-α-R	5′-AAGAACCAACGTGGGCATTG-3′

## Data Availability

All data are available from the corresponding author by request.
